# A Rare Case of Lateral Sinus Thrombosis with Carotid Space Abscess

**DOI:** 10.1155/2012/165987

**Published:** 2012-10-24

**Authors:** Gautam Bir Singh, Anil K. Rai, Sarvejeet Singh, Mukul Sinha

**Affiliations:** ^1^Department of Otorhinolaryngology and Head-Neck Surgery, Lady Hardinge Medical College and Associated Hospitals, New Delhi 110001, India; ^2^VMMC & Safdarjung Hospital, New Delhi 110029, India; ^3^Department of Otorhinolaryngology and Head-Neck Surgery, Vardhman Mahavir Medical College & Safdarjung Hospital, New Delhi 110029, India; ^4^Department of Radioimaging & Diagnosis, Vardhman Mahavir Medical College & Safdarjung Hospital, New Delhi 110029, India

## Abstract

This case report describes a case of carotid space abscess secondary to lateral sinus thrombosis associated with internal jugular vein thrombosis. With this case, we illustrate a rare entity that presented in an extremely rare manner. To the authors knowledge such a case has not been previously reported.

## 1. Introduction

Lateral sinus thrombosis (LST) is a rare complication following suppurative otitis media; further internal jugular vein (IJV) thrombosis due to LST is an extremely rare occurrence in today's modern era of medicine [1–3]. This is attributed to general awareness regarding ear discharge among masses with prompt medical treatment of it by highly potent antibiotics. However, the vague and nonspecific clinical presentation of LST still poses a medical dilemma to otolaryngologists' worldwide. With this background, we report a rare clinical presentation of LST associated with IJV thrombosis and carotid space abscess, hitherto unreported in the medical literature. The case poses a wide array of interesting diagnostic and clinical questions.

## 2. Case Report

**Figure 1:**
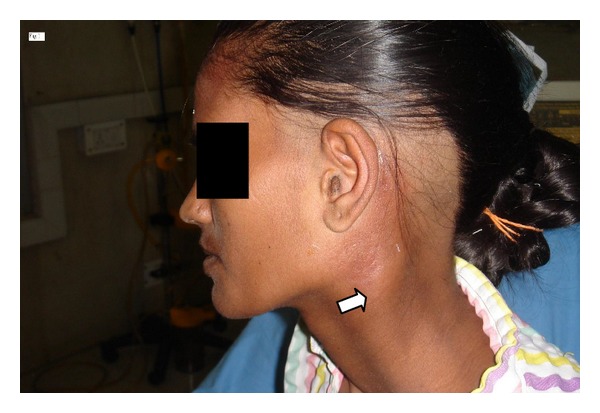
Photograph of the patient showing upper neck swelling.

**Figure 2:**
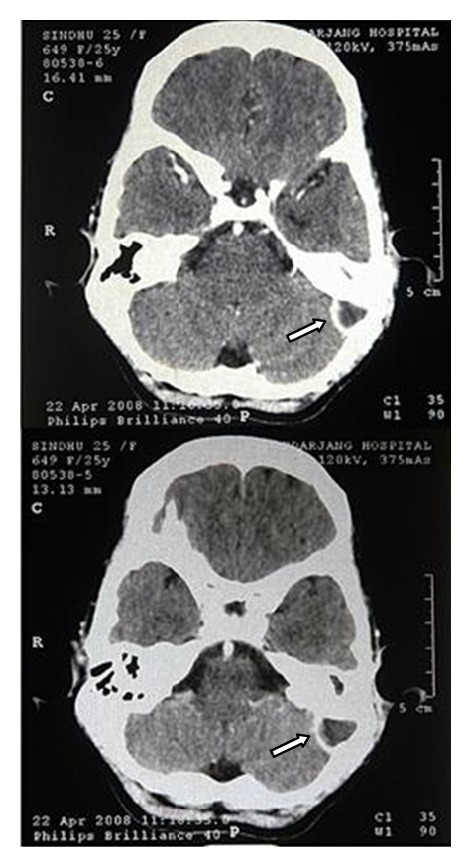
CT scan showing lateral sinus thrombosis.

A 25 years old female was referred to us from the Neurosurgical Department of our institution: Vardhman Mahavir Medical College & Safdarjung Hospital, New Delhi, India (a tertiary care central government university teaching hospital) with the diagnosis of chronic suppurative otitis media (CSOM) with lateral sinus thrombosis and swelling in the left upper part of neck. The chief complaints of the patient were ear discharge (off and on) and hearing loss in left ear since childhood and a swelling in left upper part of neck since 5 days (Figure 1). About 10 days back patient had developed vertigo, nausea and vomiting (N/V), fever with neck rigidity, and marked headache, along with left ear discharge. She consulted a private nursing home and was treated for suspected intracranial complication(? meningitis)with intravenous antibiotics. Although patients fever and N/V did settle down, mild headache persisted and soon drowsiness and lethargy supervened along with left upper neck swelling. She was then referred to our institution and after registration with the Emergency Department; the case was transferred to Neurosurgical Department. Subsequently CT scan of the patient revealed LST extending into the left jugular vein (Figure 2).

**Figure 3:**
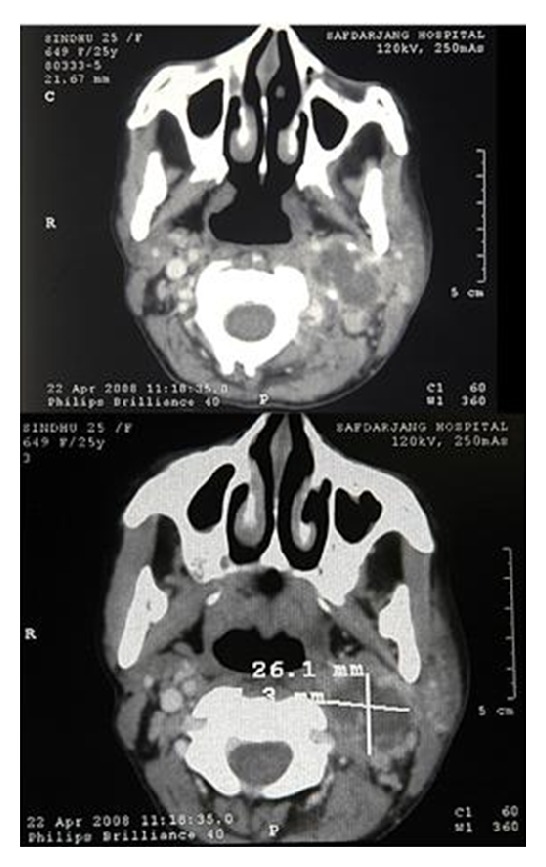
CT scan showing IJV thrombosis with carotid abscess.

The examination of the left ear after cleaning the discharge revealed a posterior-superior quadrant perforation, with erosion of the adjoining scutum and cholesteatomal flakes. Papilloedema and torticollis were absent. “Cord Sign” was present: an induration corresponding to the course of the IJV beneath the anterior border of the sternocleidomastoid muscle, though considered typical for IJV thrombosis, is rarely present [4]. The neck swelling was presumed to be an indurate mass associated with thrombosed IJV or an extension of bezolds abscess. However, CT scan neck delineated an abscess along left IJV, extending in left carotid space displacing the great vessels (Figure 3). Blood culture and the pus culture from the ear discharge were sterile. The study of hypercoagubilty status was normal.

With the final diagnosis of CSOM-left ear with LST and IJV thrombosis and carotid space abscess-left side, the patient was maintained on intravenous antibiotics-coamoxyclav and metrogyl. As the patient's condition was stable, surgical intervention in the form of a modified radical mastoidectomy (canal wall down procedure) with tympanoplasty type III along with sinus exploration was done under general anesthesia. The operative findings were as follows.

 Cholesteatoma was seen in the mastoid antrum, aditus, and attic with extension in the posterior-superior quadrant of middle ear extending well into sinus tympani and facial recess. Long process of incus was necrosed with partial erosion of handle of malleus, stapes was intact. Sinus plate was thin and eroded, once lifted with perichondrial elevator: frank pus was seen (perisinus abscess). Sinus wall was intact. A no. 18 gauge needle with 10 cc syringe was used for aspiration of the sinus. Frank pus admixed with blood was aspirated approximately amounting to 25 cc (this led to dramatic decrease in the upper neck swelling). No haemorrhage of any type was seen.

**Figure 4:**
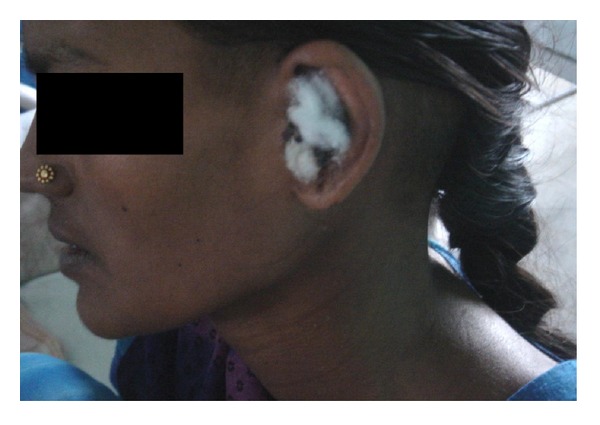
Photograph of the patient at discharge with no neck swellin.

A repeat CT scan neck revealed no abscess in the carotid space. Intravenous antibiotics were continued after surgery till discharge on the 10th postoperative day (Figure 4). Postoperative period was uneventful and the patient was kept on a regular monthly followup for a period of 3 months thereafter, with no untoward incident to report. It would be pertinent to note that treatment protocol did not include anticoagulant therapy, IJV ligation, or an external incision for drainage of the carotid abscess.

## 3. Discussion

Although the occurrence of LST has declined considerably with the advent of new genre of highly efficacious antibiotics, this sinister condition is still associated with a mortality and morbidity of 10% and 30%, respectively [2, 3]. Aetiopathogenesis is primarily attributed to the spread of infection to sigmoid sinus through a coalescent or cholesteatomal bone erosion causing formation of perisinus abscess. Subsequently adherence of fibrin, blood cells, and platelets leads to mural thrombus organization, which can cause obliteration of the sinus. LST can also be caused by an osteothrombophlebitis phenomenon. This is seen in patients with acute suppurative otitis media (ASOM) and the sinus plate is intact in such cases. Also, two distinct clinical presentations are seen—septic (with clear signs of osteomyelitis and rarely complicated by cerebral abscess) and aseptic (associated with endocrine hypertension and possible ocular signs). Delta sign (central nonenhancing clot surrounded by enhancing dural sinus wall) is regarded characteristic for LST and is delineated well by CT scan. This finding is more sensitively demonstrated by MRI. However, this sign is only 30% sensitive and is not pathognomic for sinus thrombosis (it was absent in this case too) [5]. MR venography now supercedes all other investigations for the identification of the thrombus in the sigmoid sinus as evidenced by flow void [6]. Despite the advantages of MRI, its cost and selective availability especially in developing countries limit its use. It is thus mandatory only in those suspicious cases where the performed CT scan fails to demonstrate the thrombus [7].

The present case in focus brings forth many interesting features of lateral sinus thrombosis highlighting the changing clinical face of the said lesion. This case report delineates an extended complication such as deep neck infections (carotid space infection in this case) following lateral sinus thrombosis. This is probably due to reactionary inflammatory response of the thrombosed and infected IJV. It is pertinent to note that in young adults, LST is now more often seen in association with generalized hypercoaguble state, inherited or acquired [8]. However, this condition was absent in our case. Yet another important factor responsible for LST secondary to ASOM and CSOM is antibiotic resistance [9]. Whether this clinical record can be attributed to antibiotic resistance or protracted course of the ear disease, or both, the subject is open to debate.

Further, it is important to note that the said deep neck abscess was drained via the infected sinus without an external incision. This therapeutic approach resulted in a good outcome in our case, thus an external incision for drainage of deep neck abscess was omitted. The patient also responded well to the mastoid surgery and post operative antibiotic treatment, thereby making IJV ligation unnecessary. In this context it would be prudent to note that in modern otology, IJV ligation is reserved for persistent septicemia even after mastoidectomy or septic pulmonary or extra pulmonary embolization [10]. We treated this patient successfully without any use of anticoagulants. The use of anticoagulants in LST is controversial [2, 3, 5]. The general consensus is that concomitant use of anticoagulants with antibiotics is best avoided in septic conditions as it leads to dissemination of the emboli. Moreover, the absence of hypercoaguble state in the said case also prompted us to eliminate anticoagulants all together from the management protocol. The medical literature, however, cites the importance of this modality of treatment to prevent complications attributed to thrombus persistence and its possible propagation [2, 11]. Hence, whether the early prophylactic use of anticoagulant therapy could have averted the said carotid space infection, secondary to IJV thrombosis is speculative.

The authors would best define the presentation of LST as subtle since the introduction of antibiotics. Along with non-specific intracranial signs and symptoms, the neck swelling was an ominous sign of advanced stage of lateral sinus thrombosis, obviously missed by all treating physicians out of ignorance. This rare clinical record thus emphasizes the importance of heightened awareness of the changing presentation of complication of LST, so that delayed diagnosis as a result of misdiagnosis is avoided in this life threatening complication.

In a literature search using Medline services/PubMed database using the medical subject function, authors could find no such case of lateral sinus thrombosis associated with deep neck abscess as described here in. In summary, the unusual clinical presentation and management of the carotid space infection as a result of LST with no change in coagulation profile of the patient make this case report unique and prompted us to share our professional experience with the medical fraternity.
